# Inhibition of the prolyl isomerase Pin1 improves endothelial function and attenuates vascular remodelling in pulmonary hypertension by inhibiting TGF-β signalling

**DOI:** 10.1007/s10456-021-09812-7

**Published:** 2021-08-11

**Authors:** Kondababu Kurakula, Quint A. J. Hagdorn, Diederik E. van der Feen, Anton Vonk Noordegraaf, Peter ten Dijke, Rudolf A. de Boer, Harm Jan Bogaard, Marie José Goumans, Rolf M. F. Berger

**Affiliations:** 1 Department of Cell and Chemical Biology, Leiden University Medical Center, Einthovenweg 20, 2333 ZC Leiden, The Netherlands; 2 Department of Paediatric Cardiology, Beatrix Children’s Hospital, Center for Congenital Heart Diseases, University Medical Center Groningen, University of Groningen, Groningen, The Netherlands; 3 Department of Pulmonary Medicine, Amsterdam Cardiovascular Sciences, Amsterdam UMC, Vrije Universiteit Amsterdam, Amsterdam, The Netherlands; 4 Department of Cell and Chemical Biology, Oncode Institute, Leiden University Medical Center, Leiden, The Netherlands; 5 Department of Cardiology, University Medical Center Groningen, University of Groningen, Groningen, The Netherlands

**Keywords:** Pulmonary arterial hypertension, Vascular remodelling, Endothelial cell, TGF-β/BMP signalling, Inflammation

## Abstract

Pulmonary arterial hypertension (PAH) is a devastating disease, characterized by obstructive pulmonary vascular remodelling ultimately leading to right ventricular (RV) failure and death. Disturbed transforming growth factor-β (TGF-β)/bone morphogenetic protein (BMP) signalling, endothelial cell dysfunction, increased proliferation of smooth muscle cells and fibroblasts, and inflammation contribute to this abnormal remodelling. Peptidyl-prolyl isomerase Pin1 has been identified as a critical driver of proliferation and inflammation in vascular cells, but its role in the disturbed TGF-β/BMP signalling, endothelial cell dysfunction, and vascular remodelling in PAH is unknown. Here, we report that Pin1 expression is increased in cultured pulmonary microvascular endothelial cells (MVECs) and lung tissue of PAH patients. Pin1 inhibitor, juglone significantly decreased TGF-β signalling, increased BMP signalling, normalized their hyper-proliferative, and inflammatory phenotype. Juglone treatment reversed vascular remodelling through reducing TGF-β signalling in monocrotaline + shunt-PAH rat model. Juglone treatment decreased Fulton index, but did not affect or harm cardiac function and remodelling in rats with RV pressure load induced by pulmonary artery banding. Our study demonstrates that inhibition of Pin1 reversed the PAH phenotype in PAH MVECs in vitro and in PAH rats in vivo, potentially through modulation of TGF-β/BMP signalling pathways. Selective inhibition of Pin1 could be a novel therapeutic option for the treatment of PAH.

**Supplementary Information:**

The online version contains supplementary material available at 10.1007/s10456-021-09812-7.

## Introduction

Pulmonary arterial hypertension (PAH) is a progressive disorder in which endothelial dysfunction and vascular remodelling obstruct small pulmonary arteries. This results in a marked and sustained elevation of pulmonary artery (PA) pressure, and eventually right ventricular (RV) failure and death [1–4]. The abnormal pulmonary vascular remodelling is characterized by a hyperproliferative, apoptosis-resistant and inflammatory phenotype of pulmonary arterial endothelial cells (PAECs) and smooth muscle cells (SMCs) [5–9]. The pathophysiologic mechanism involves several signalling pathways, including the TGF-β/BMP pathway [10–13]. Despite recent advances in the molecular understanding of the vascular remodelling in PAH, current therapies fail to reverse this vascular remodelling, resulting in only a modest improvement in morbidity and mortality. Therefore, there remains an urgent need to identify new molecular targets that can reverse vascular remodelling to develop effective and safe treatments for PAH patients.

We and others have previously shown that the peptidyl-prolyl isomerase Pin1 acts as a critical driver of vascular cell proliferation, apoptosis and inflammation, and is implicated in several cardiovascular diseases such as atherosclerosis, coronary restenosis, and cardiac hypertrophy [14–16]. Pin1 regulates endothelial nitric oxide synthase and induces endothelial dysfunction [17]. Moreover, Pin1 inhibitor juglone prevents diabetes-induced endothelial dysfunction via NF-κB signalling [18]. In contrast, Pin1 knockout mice exhibited increased aortic endothelial nitric oxide synthase, endothelial dysfunction, and hypertension [19]. Pin1 belongs to the parvulin subfamily of peptidyl-prolyl cis–trans isomerase (PPIase) group of proteins. Pin1 is the only PPIase that specifically binds phosphorylated Ser/Thr-Pro protein motifs and catalyzes the cis/trans isomerization of the peptide bond [20–23]. Through protein–protein interactions and inducing conformational changes on the substrates, Pin1 regulates diverse cellular processes. Pin1 has been shown to modulate signal transduction by interacting with a diversity of transcription factors [20, 24–27]. Interestingly, Pin1 has been reported to interact with TGF-β/BMP-specific receptor-regulated transcription factors Smad1, Smad2, and Smad3 but not with the common mediator Smad Smad4 [28]. Pin1 activity has been shown to be essential for skeletal muscle fusion through structural modification of Smad3 in the linker region [29]. Furthermore, a positive feedback loop of TGF-β1/promyelocytic leukaemia SUMOylation/Pin1 has been shown to promote the cardiac fibrosis [30]. FOXM1 and PLK1, two transcription factors able to modulate TGF-β signalling and shown to be involved in PAH pathogenesis [31, 32], are also substrates of Pin1. Although Pin1 interacts with TGF-β/BMP dependent Smad proteins, a function for Pin1 in the disturbed TGF-β/BMP signalling and vascular remodelling in PAH has not been reported to date.

Given the effect Pin1 has on proliferation, apoptosis and inflammation of ECs and SMCs, and its effects on several known PAH related signalling pathways, including the TGF-β/BMP cascade, we hypothesized that Pin1 is involved in PAH pathophysiology. Here, we show that inhibition of Pin1 decreases proliferation, inflammation, and TGF-β signalling in pulmonary microvascular ECs in vitro. Chronic oral administration of the Pin1 inhibitor juglone reversed abnormal vascular remodelling, without affecting RV function in a rat model of PAH nor in a rat model of isolated RV pressure loading. To our knowledge, this is the first time a role for Pin1 in PAH was demonstrated and suggests that selective inhibition of Pin1 represents a novel therapeutic target in PAH.

## Methods

Please see the supplementary material for detailed methods.

### Cell culture and tissue sections

Collection of lung specimens was approved by the local ethical committee and written informed consent from patients was obtained. Human pulmonary artery microvascular endothelial cells (MVECs) and smooth muscle cells (PASMCs) were isolated and cultured from idiopathic PAH patients and control lung explant tissue as previously described [33, 34].

### Juglone treatment in the MCT + Shunt rat PAH model

All animal experiments were approved by the Dutch Central Ethical Committee for Animal Experiments and the Animal Care Committee of the University Medical Center Groningen and were carried out in compliance with guidelines issued by the Dutch government. All experiments were conducted according to published standards for preclinical and translational research in PAH [35]. The MCT + Shunt (MS) rat PAH model was used to study pulmonary vascular remodelling in 25 male Wistar rats and was performed as described previously [36]. Rats were randomly assigned to 3 groups: (1) MCT + Shunt sacrificed at T21 (MS21) as a baseline group; (2) treatment with vehicle (5% DMSO in drinking water) from T21, with sacrifice at T35 (MS35Veh); (3) treatment from T21 with 5 mg/kg juglone in vehicle (5%DMSO in drinking water) with sacrifice at T35 (MS35Juglone). Echocardiography was also performed before the treatment (at day 21) started to determine baseline cardiac function. All measurements and analyses were done in a blinded manner.

### Juglone treatment in the PAB rat RV pressure load model

To assess direct myocardial effects of juglone in this setting, isolated RV pressure load was created in 16 male Wistar rats by main pulmonary artery banding (PAB) surgery. Rats were randomly assigned to (1) treatment with vehicle (5% DMSO in drinking water) from T28 with sacrifice at T56 (PABveh56), or (2) treatment with 5 mg/kg Juglone in vehicle (5% DMSO in drinking water) from T28 with sacrifice at T56. Before sacrifice, all rats underwent haemodynamic evaluation by echocardiography, after which lungs and hearts were collected for histopathologic evaluation.

### Quantitative pulmonary vascular morphometry

40 vessels (diameter < 50 µm) per lung were analysed according to a standardized pulmonary vascular morphometry protocol, described in detail previously [36, 37].

### Statistical analysis

Statistical analyses were performed using the GraphPad Prism software for windows, version 7.0. The mean value (± SD) was calculated for all samples, and significance was determined by either the unpaired *t*-test or analysis of variance (one- way ANOVA). Bonferroni multiple comparison test was applied to correct for multiple testing. A value of *P* < 0.05 was considered significant.

## Results

### Pin1 expression is increased in PAH

**Fig. 1:**
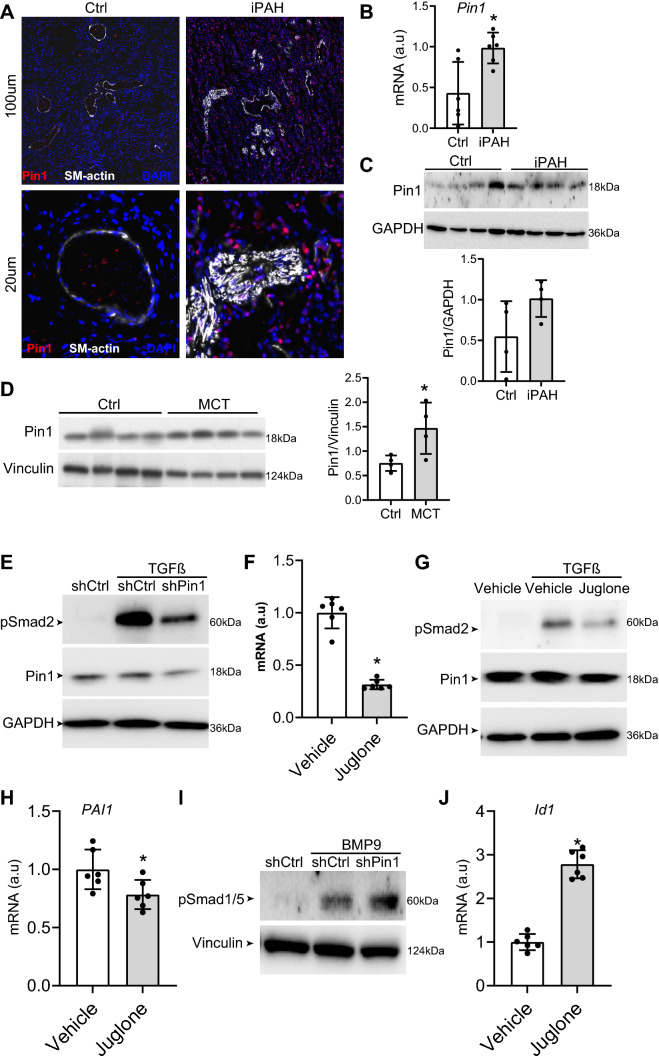
Pin1 expression is increased in PAH and Pin1 inhibition modulates TGFβ/BMP signalling in microvascular ECs. **A** Representative immunofluorescence photomicrographs of Pin1 (red) and α-smooth muscle actin (SM-actin, white) in human pulmonary arteries from control and iPAH lungs (*n* = 5). DAPI (blue). **B** qRT-PCR was performed to assess mRNA expression of Pin1 in MVECs from control and iPAH patients (*n* = 6 per group). **C**) Representative western blots with relative densitometric analyses showing Pin1 in MVECs from control and iPAH patients (*n* = 4 per group). **D** Representative western blots with relative densitometric analyses showing Pin1 in total lungs from MCT-induced PAH rats (*n* = 4 per group). **E** Representative western blots showing pSmad2 in MVECs following knock-down of Pin1 by shPin1 lentivirus. **F** qRT-PCR was performed to assess mRNA expression of Pin1 in MVECs, following treatment with Pin1 inhibitor, juglone. **G** Representative western blots showing pSmad2 in MVECs, following treatment with juglone. **H** qRT-PCR was performed to assess mRNA expression of PAI-1 in MVECs, following treatment with juglone (*n* = 6). **I** Representative western blots showing pSmad1/5 in PASMCs following knock-down of Pin1 by shPin1 lentivirus. **J** qRT-PCR was performed to assess mRNA expression of Id1 in MVECs, following treatment with juglone (*n* = 6). *P < 0.05. Error bars, mean ± SD

To determine if Pin1 is involved in the pathology of PAH we first performed immunofluorescent analysis of lung of idiopathic (iPAH) patients and showed that the expression of Pin1 is increased when compared to controls (Fig. 1A). Increased expression of Pin1 was also found in the rat lungs of MCT-, SuHx-, and MCT + shunt-induced PAH (Supplementary Fig. S1). In cultured human pulmonary MVECs, both mRNA and protein levels of Pin1 were modestly increased in iPAH compared to controls (Fig. 1B, C). Consistent with these findings, protein levels of Pin1 were significantly increased in the total lung lysates of the MCT-induced rat model of PH (Fig. 1D). In summary, Pin1 levels are increased in lungs and pulmonary MVECs of iPAH patients.

### Pin1 modulates TGF-β/BMP-SMAD signalling in MVECs

It is well accepted that disturbed TGF-β/BMP signalling plays a crucial role in the development and progression of PAH [10, 38], and several Smad proteins are substrates of Pin1 (Table 1). Therefore, we aimed to investigate if and how Pin1 influences TGF-β/BMP-SMAD signalling in MVECs. Interestingly, inhibition of Pin1 expression by shRNA (Supplementary Fig. S2A) significantly inhibited TGFβ-induced phosphorylation of Smad2 and Smad3 (pSmad2/3) (Fig. 1E).[10] Inhibiting Pin1 isomerase activity with juglone (Fig. 1F) also decreased TGFβ-induced phosphorylation of Smad2/3 in MVECs (Fig. 1G). Furthermore, juglone attenuated the mRNA levels of PAI1, a downstream target gene of TGFβ signalling in MVECs (Fig. 1H). In addition, knock-down of Pin1 also decreased expression of pSmad2/3 in PASMCs (Supplementary Fig. S2B).

**Table 1:** pSer/Thr-Pro motif analysis of known Pin1 substrates and TGF-β/BMP signalling components

Protein	Known Pin1 substrate	Proline-rich region	pSer/Thr-Pro Motif	Domains and other protein regions	Pin1 Substrate
BMPR2	No	–	–	PK ATP-binding region (aa. 209–230). Thr-rich region profile (aa. 603–643)	No
ID1	No	–	–	Myc-type basic helix-loop-helix domain	No
SMAD1	Yes [28, 50]	(aa.164–252)	pSer-Pro (aa.171- 174)	MH1 (aa. 112–136) and MH2 (aa. 271–465)	Yes
SMAD5	Yes [28, 50]	(aa.165–229)	pSer-Pro (aa.172–175)	MH1 (aa.13–137) and MH2 (aa.271–465)	Yes
SMAD8/9	Yes [28, 50]	–	pSer-Pro (aa.176–179)	MH1 (aa.16–140) and MH2 (aa.236–430)	Yes
SMAD2	Yes [28, 50, 51]	–	pSer-Pro (aa.245–248)	MH1 (aa.10–176) and MH2 (aa.274–467)	Yes
SMAD3	Yes [28, 50]	–	pThr-Pro motif (aa.: 179–180)	MH1 (aa.10–137) and MH2 (aa.232–425)	Yes
ACVR2A Isoform I	No	–	–	PKdomain (aa.192–485) and Ser/Thr active site (aa. 318–330)	No
ACVR2A Isoform 2	No	–	–	PPK domain (aa.84–377) and Ser/Thr active site (aa. 210–222)	No
ACVR2B	No	–	–	PPK domain (aa.190–480) and Ser/Thr active site (aa.317–329)	No
CDC25c	Yes [52]	–	pThr-Pro (aa.:67–69)	Rodanese domain (aa. 321–428) and active site (aa. 377)	Yes
Microtubule-associated Tau Isoform 1	Yes [52]	–	pSer-Pro (aa.: 228–231)	Tau and MAP proteins tubulin-binding repeats (aa: 561–591, 592–622, 623–653, 654–685)	Yes
Cyclin D1	Yes [14, 51]	–	pThr-Pro motif (aa.: 286–289)	Cyclin signature	Yes
Nur77 (NR4A1 Isoform 1)	Yes [14]	–	pSer-Pro motif (aa.: 152–155)	Ser-rich region (aa.79–164). Nuclear hormone receptor DBD (aa. 264–339) and signature (aa. 267–293)	Yes

Motif analysis data represents results obtained from PROSITE (Swiss Institute of Bioinformatics, Lausanne, Switzerland). *Column 1* (Protein) represents all analysed proteins. *Column 2* (Literature) shows whether proteins are known Pin1 substrates according to published literature. *Column 3* (Pro-rich region) represents proline-rich region search (where Pin1 has more binding affinity) within the analysed proteins. *Column 4* (pSer/Thr-Pro motif) shows whether proteins contained Pin1 binding motif. *Column 5* illustrates other domains found within the protein sequences. *Column 6* depicts whether proteins are Pin1 substrates. *aa.* amino acid numbers, *PK* Protein Kinase, *DBD* DNA-binding Domain

To investigate the effect of Pin1 on canonical BMP signalling, we determined the level of Smad1/5/8 phosphorylation upon knock-down of Pin1 and observed that reduced Pin1 levels markedly increased the phosphorylation of Smad1/5/8 upon BMP9 stimulation in PASMCs (Fig. 1I). In line with this, juglone augmented the expression levels of Id1 in PAH MVECs, a downstream target gene of BMP signalling. Moreover, Pin1 over-expression decreased whilst juglone enhanced BMP9-induced BMP/SMAD reporter (BRE-luc) activity in PAH MVECs (Supplementary Fig. S2C, D) [39]. Finally, under full serum and TNFα-stimulated conditions, knock-down of Pin1 enhanced BMPR2 and Id3 expression as demonstrated by western blot analysis (Supplementary Fig. S2E). Pin1 over-expression did not influence BMPR2 stability in HEK293T cells (Supplementary Fig. S2F–H), suggesting that Pin1 might directly enhance the BMPR2 expression. Taken together, our data demonstrated that inhibition of Pin1 decreases TGF-β/SMAD2/3 signalling whilst increases BMP/SMAD1/5 signalling in MVECs.

### Juglone attenuates cell viability and proliferation of MVECs and PASMCs

**Fig. 2:**
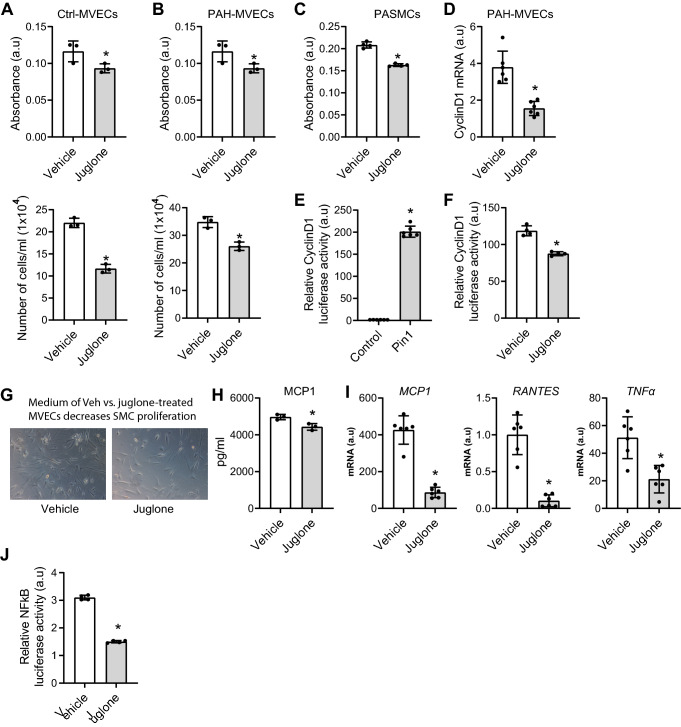
Pin1 modulates proliferation and inflammation of MVECs. **A**, **B** MTT (upper panel) and cell count (lower panel) assays were performed to assess proliferation of control (**A**) and PAH (**B**) MVECs following treatment with juglone (*n* = 3). **C** MTT assays were performed to assess proliferation of healthy PASMCs following treatment with juglone (*n* = 3). **D** qRT-PCR was performed to assess mRNA expression of CyclinD1 in PAH MVECs following treatment with juglone (*n* = 6). **E**, **F** CyclinD1 promoter luciferase activity in HEK293T cells was measured following ectopic expression of Pin1 (**E**) and treatment with juglone (**F**). **G** SMCs cultured in medium conditioned by PAH MVECs treated with vehicle or juglone. **H** ELISAs for MCP-1 were performed using supernatants from MVECs following treatment with juglone and stimulation with TNFα for 6 h (*n* = 3). **I** qRT-PCR was performed to assess mRNA expression of MCP-1, RANTES, and TNFα following treatment with juglone and stimulation with TNFα for 6 h (*n* = 6). **J** TNFα-induced NFκB-luciferase activity in HEK293T cells was measured following treatment with juglone and stimulation with TNFα for 6 h (*n* = 6). **P < 0.05. Error bars, mean ± SD

Next, we explored whether inhibition of Pin1 by juglone normalizes the hyper-proliferative status of PAH MVECs and observed that Juglone reduced cell viability and proliferation of both PAH and control MVECs (Fig. 2A, B). Juglone also inhibited the cell viability of PASMCs (Fig. 2C). Since Pin1 is reported to induce cell proliferation by increasing the expression of CyclinD1 [40], we found that juglone decreased the mRNA levels of CyclinD1 (Fig. 2D), a key mediator of cell proliferation. Consistent with this, over-expression of Pin1 increased (Fig. 2E) and inhibition of Pin1 activity by juglone decreased CyclinD1 promoter activity in HEK293T cells (Fig. 2F). We further showed that medium conditioned by PAH MVECs induced proliferation of normal SMCs, whereas juglone treatment of PAH MVECs resulted in conditioned medium that decreased SMC proliferation (Fig. 2G), signifying that juglone modulates the pro-proliferative crosstalk of ECs to SMCs. Taken together, these data suggest that Pin1 drives PAH MVEC proliferation.

### Juglone inhibits inflammation of MVECs by inhibiting NFκB activity

We next investigated the effect of juglone on the pro-inflammatory status of PAH MVECs. Juglone significantly decreased TNFα-induced expression of MCP-1 at protein level (Fig. 2H) and inhibited the expression levels of MCP-1, RANTES, and TNFα at mRNA level (Fig. 2I) in PAH MVECs. Furthermore, juglone strongly decreased whilst overexpression of Pin1 markedly increased the transcriptional activity of the NFκB promoter (Fig. 2J; Supplementary Fig. S3A). Finally, we also found that juglone decreases expression of endothelin1, a known NFκB target gene [41] (Supplementary Fig. S3B). Altogether, these data suggest that inhibition of Pin1 attenuates the pro-inflammatory response of PAH MVECs through inhibition of the NFκB pathway.

### Juglone treatment reverses the development of PAH, attenuates inflammation, and inhibits TGF-β signalling in vivo

**Fig. 3:**
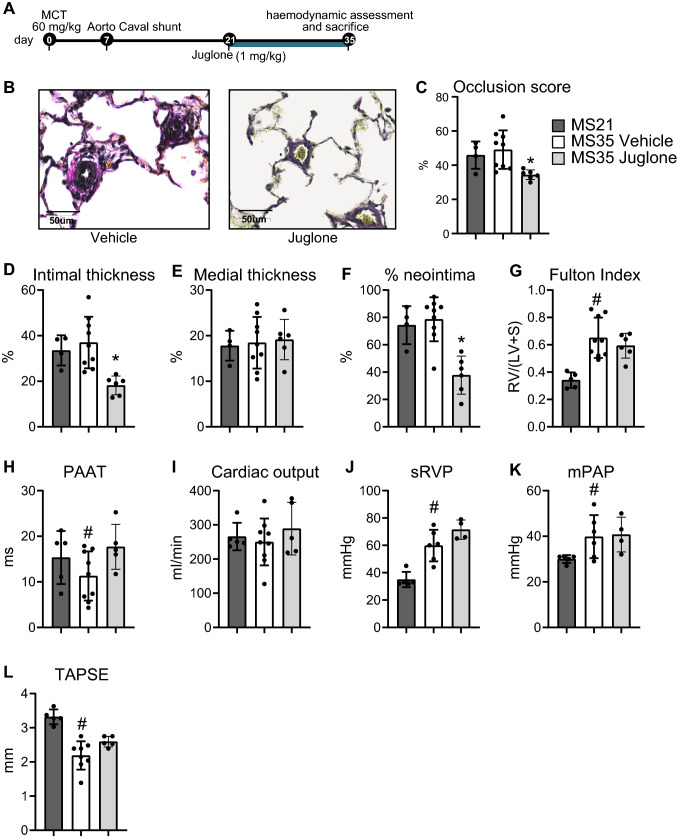
Juglone reverses pulmonary vascular remodelling in vivo. **A** Experimental design for the in vivo intervention study with juglone in MCT-shunt rats (MS-PAH). **B** EvG staining. representative examples of vascular lesions. Scale bars, 50 um. **C**–**F** Quantification of vascular occlusion (**C**), intimal thickness (**D**), medial thickness (**E**), and % of the neointimal lesions (**F**). **G**–**I** Quantification of haemodynamics: Fulton index (**G**), pulmonary artery acceleration time (PAAT; **H**), and cardiac output (CO, **I**). *n* = 4–9 per group. **P < 0.05. Error bars, mean ± SD. *Veh * vehicle (5% DMSO)

Since our in vitro data suggest that inhibiting Pin1 with juglone has therapeutic potential in the treatment of PAH, we induced PAH in rats using the MCT + Shunt model and treated them with juglone (1 mg/kg/day) orally in drinking water from 21 days onwards after the induction of PAH (Fig. 3A). As can be appreciated, the vascular occlusion score, intimal thickness,  medial thickness, and % of vessels with neointima formation were comparable in the MS21 and MS35veh rats (Fig. 3B–F), indicating that treatment was initiated at a time point (21 days) at which advanced pulmonary vascular disease had developed. Juglone significantly reduced vascular occlusion (Fig. 3C), intimal thickness (Fig. 3D), and % of neointima (Fig. 3F) compared to vehicle-treated rats after 14 days of treatment. We did not observe an effect of juglone on medial thickness (Fig. 3E). Pulmonary artery acceleration time (PAAT), an indirect measure of pulmonary vascular resistance, was significantly improved in juglone-treated rats, when compared to vehicle-treated rats (Fig. 3H). However, juglone had no effect on the Fulton index [RV/(LV + IVS) weight ratio] and cardiac output (CO) (Fig. 3G, I). In conclusion, oral treatment with juglone reversed abnormal vascular remodelling and improved PAAT in MS-PAH rats.

**Fig. 4:**
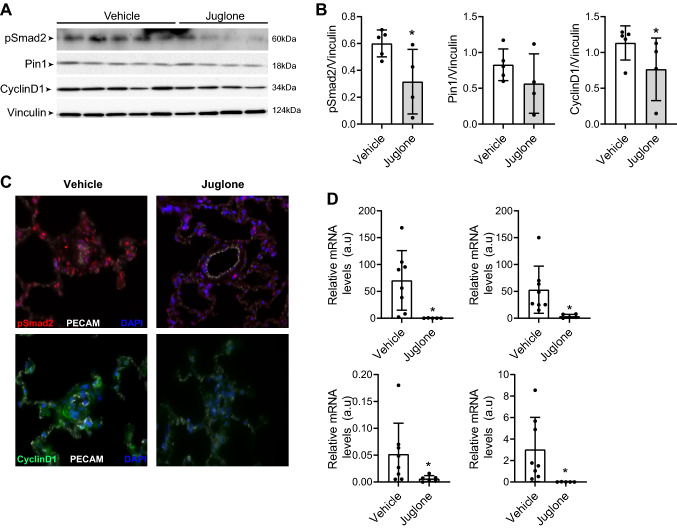
In vivo effects of juglone on TGF-β signalling, proliferation, and inflammation. **A**,** B** Representative western blots with relative densitometric analyses showing pSmad2, Pin1, and CyclinD1 in whole-lung lysates of vehicle and juglone-treated MS-rats (*n* = 4–5/group). Vinculin served as loading control. **C** Representative immunofluorescence photomicrographs of pSmad2 (red, upper panel) and CyclinD1 (green, lower panel) in vehicle and juglone-treated MS-rats (*n* = 6/group). PECAM (white) and DAPI (Blue). **D** qRT-PCR was performed to assess mRNA expression of VCAM-1, ICAM-1, MCP-1, and CCL5 in vehicle and juglone-treated MS-rats (*n* = 5–8/group). **P < 0.05. Error bars, mean ± SD. *Veh *vehicle (5% DMSO)

Since Pin1 modulates TGF-β signalling [28], we next examined the effect of juglone treatment on TGF-β signalling in the lungs of the MS-rats. We found that juglone significantly decreased the expression levels of pSmad2/3 as demonstrated by western blot analysis (Fig. 4A, B), which was confirmed using immunofluorescent analysis (Fig. 4C). Juglone slightly decreased the expression levels of Pin1 and CyclinD1 in the western blot analysis (Fig. 4A, B). However, immunofluorescent analysis shows that juglone decreased expression levels of CyclinD1 (Fig. 4C). Finally, juglone inhibited the expression levels of endothelial adhesion molecules and pro-inflammatory cytokines such as VCAM-1, ICAM-1, CCL5, and MCP-1 (Fig. 4D). Altogether, juglone reduced TGF-β signalling, decreased cell proliferation, and attenuated inflammation, at least partly, in vivo via Pin1.

### Juglone does not harm RV function during increased pressure load

**Fig. 5:**
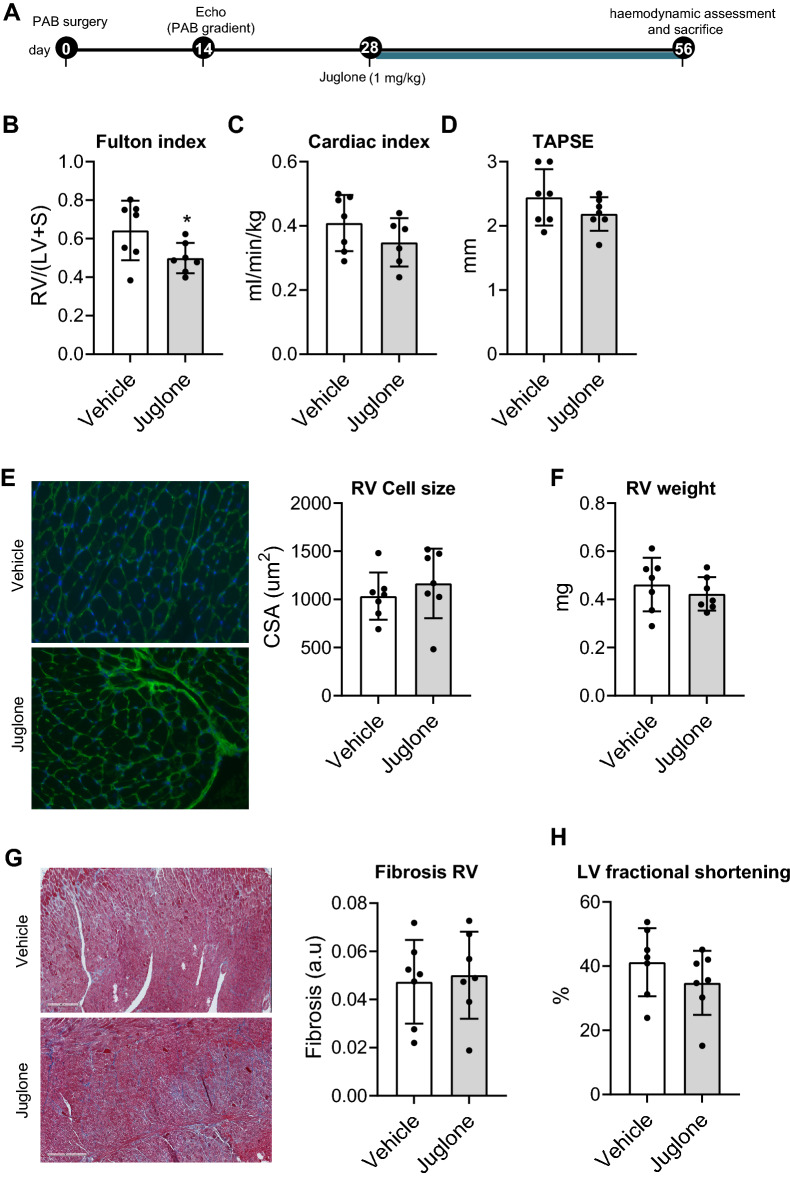
Juglone has no direct cardioprotective effect. **A** Experimental design for the in vivo intervention study with juglone in the rat pulmonary artery banding (PAB) model for isolated RV pressure load. **B**–**D** Quantification of haemodynamics: Fulton index (**B**), Cardiac index (**C**), and TAPSE (**D**). **E** Wheat germ agglutinin staining for cardiomyocyte cross-sectional area measurement and quantification. **F** Weight of the RV. **G** Masson staining for fibrosis measurement. (H) LV fractional shortening. *n* = 7 per group. **P < 0.05. Error bars, mean ± SD. *Veh* = vehicle (5% DMSO), *CSA *cross-sectional area measurement, *CI *cardiac index, *RV/(LV/IVS) *right ventricular to left ventricular/intraventricular septal weight ratio, *TAPSE *tricuspid annular plane systolic excursion

**Fig. 6:**
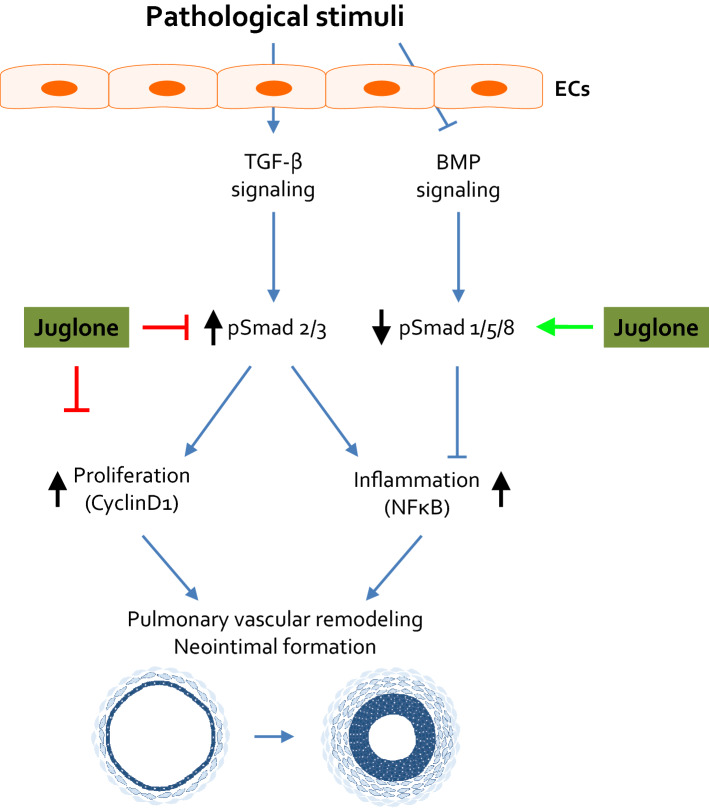
Proposed mechanism of Pin1 in the reversal of PAH. Juglone inhibits Pin1 activity which (i) blocks proliferation through inhibition of cell cycle proteins; (ii) inhibits inflammation through blocking the NFκB pathway; (iii) inhibits TGF-β signalling via pSmad2/3 and enhances BMP signalling via pSmad1/5/8-Id1/3 axis. Abnormal proliferation and excessive inflammation along with enhanced TGF-β signalling and impaired BMP signalling leads to initiation and progression of PAH. Inhibition of Pin1 with selective Pin1 inhibitors might reverse the abnormal remodelling and inhibits PAH

We next studied the direct effects of juglone treatment on RV remodelling in the setting of isolated RV pressure load, induced by PAB in rats. At day 14, we measured PAB pressure gradient using echocardiography to confirm effective and equal pressure load at baseline. In both groups, the pressure gradient equally increased from day 14 to day 56, indicating adaptation to pressure load (Supplementary Figure S4A). Although juglone decreased Fulton index, cardiac index, tricuspid annular plane systolic excursion (TAPSE), LV fractional shortening, RV hypertrophy, RV fibrosis, the number of capillaries in both LV and RV, and the ratio of capillaries to myocytes both in LV and RV were not affected by juglone (Fig. 5, Supplementary Figure S4). Finally, we did not observe an effect of juglone on the haemoglobin and platelet count. A statistically significant, but clinically insignificant increase in the number of white blood cells was observed (Supplementary Figure S4F). Collectively, these data indicated that juglone does not seem to benefit nor harm cardiac function in response to increased RV pressure load, with no signs of altered or adverse remodelling. We summarized our findings in Fig. 6.

## Discussion

In this study, we identify the peptidyl-prolyl isomerase Pin1 as a novel regulator of vascular remodelling and TGF-β/BMP signalling in PAH. We demonstrate that Pin1 is up-regulated in the pulmonary vasculature in PAH, and postulate that inhibition of Pin1 isomerase activity by juglone could be a novel therapeutic option to reverse the abnormal vascular remodelling in PAH. The model we propose (Fig. 6) is based on our observations that (i) Pin1 expression is up-regulated in both pulmonary MVECs and lungs of iPAH patients; (ii) lack of Pin1 inhibits EC dysfunction and PA-SMC proliferation; (iii) inhibition of Pin1 by juglone as well as Pin1 knock-down attenuates inflammation through inhibition of the NFκB pathway; (iv) juglone inhibits MVEC dysfunction, inhibits TGF-β signalling, and potently augments BMP/SMAD signalling in MVECs in vitro and rat lungs in vivo. Furthermore, we demonstrated that (v) Pin1 inhibition reduces abnormal remodelling of the pulmonary vasculature in a rat model of neointimal PAH. Finally, in a second rat model of proximal RV pressure load, where RV remodelling occurs independently from effects on the pulmonary vasculature, (vi) Pin1 inhibition does not affect cardiac function, in the context of isolated RV pressure loading.

Pin1 has been implicated in several vascular diseases, including atherosclerosis, cardiac hypertrophy, and coronary restenosis, where Pin1 induces proliferation of endothelial cells, smooth muscle cells, and fibroblasts, whilst at the same time promotes inflammation through activation of the NFκB pathway [14–16]. Pulmonary vascular cell proliferation and inflammation are key features of pulmonary vascular remodelling in PAH [5–8]. A key mechanism by which Pin1 and its inhibitor juglone inhibit cell proliferation is through regulation of CyclinD1, a known substrate of Pin1 [40]. Indeed, in the present study we demonstrated that inhibition of Pin1 in PAH MVECs reduced CyclinD1 expression and cellular proliferation. We found that Pin1 is a key player at the interplay between these cells since the secretome of diseased PA-ECs stimulated the growth of PA-SMC. Pin1 and its inhibitor juglone inhibit EC and SMC proliferation via inhibition of the NFκB pathway, thereby reducing the production of several cytokines. Here, we confirm that juglone reduces the secretion of inflammatory cytokines in pulmonary MVECs and in lung tissue of the juglone-treated rats with PAH.

To test efficacy of juglone in vivo, we showed that oral treatment with juglone to rats with established PAH halted and even reversed the phenotype. To our knowledge, this is the first time a pathological role for Pin1 in abnormal pulmonary remodelling of PAH was shown, providing a rationale for Pin1 inhibition as a novel therapeutic strategy for PAH. Juglone as an inhibitor of Pin1 was evaluated in MCT-Shunt model of PAH, which demonstrates endothelial dysfunction, vascular remodelling, and neointimal formation similarly to human PAH. Current PAH treatments aim to relieve vasoconstriction rather than directly inhibiting pulmonary vascular remodelling and improving RV function. Here, we demonstrate that juglone reduced pulmonary vascular remodelling and that the therapeutic efficacy conferred by juglone was not likely due to vasodilation, because chronic inhibition of Pin1 did not affect systemic blood pressures and heart rate in this model. Therefore, the efficacy of Pin1 inhibition on improving the pulmonary acceleration time in vivo was likely due to attenuation of pulmonary vascular remodelling. Indeed, we found that juglone reduced vascular remodelling which was accompanied by the restoration of EC function, inhibition of TGFβ signalling, and augmented BMP signalling. Furthermore, germline mutations in BMPR2 are the strongest known genetic risk factor associated with PAH, and both BMPR2 and BMP signalling are reduced even in iPAH patients [4]. Importantly, loss of BMPR2 has been linked to increased inflammation and proliferation of pulmonary ECs, and contributes to abnormal vascular remodelling in PAH [42], and either enhanced BMP signalling or inhibition of TGF-β signalling reduced the development of PAH in pre-clinical models [43–46]. Our data provides the first in vitro and in vivo evidence that juglone inhibits increased TGF-β signalling whilst augmenting impaired BMP signalling. Our findings imply that juglone, via the modulation of TGF-β/BMP signalling, may significantly promote EC function in iPAH. Therefore, inhibition of Pin1 can serve as a novel therapeutic approach for PAH patients with augmented TGF-β signalling and impaired BMP signalling. Based on our present data, as well as literature describing a role for both TGF-β/BMP signalling pathways in ECs and SMCs, further research is warranted to dissect the mechanistic role of Pin1 on these pathways in ECs and SMCs.

Although Pin1 inhibition supports RV function in the MS-PAH rat model in vivo, any effects on the RV in this model could very well have resulted from reduced pulmonary pressure and afterload. Therefore, to examine the direct effect of Pin1 inhibition on RV function and the RV myocardium, juglone was evaluated in a rat model of isolated RV pressure load. Oral administration of juglone, starting 28 days after PAB surgery when RV dysfunction was established, did not influence RV function, demonstrating that whilst reducing pulmonary vascular remodelling, Pin1 inhibition did not benefit nor harm the RV in the context of RV pressure load. Previous studies demonstrated in animal models of LV remodelling and heart failure that juglone reduces fibrosis and improves LV function. However, juglone had no effect on the pathological RV remodelling and RV haemodynamics in the PAB model, possibly due to shorter duration of treatment, dosage, or most importantly, differences in LV vs RV remodelling mechanisms [47]. Future studies are needed to understand the role of Pin1 in the RV function.

Juglone significantly reversed abnormal vascular remodelling and increased the pulmonary acceleration time in the MS-PAH rat model. Importantly, the beneficial effect of juglone was associated with inhibition of Pin1 expression and TGF-β signalling and activation of BMP signalling. Although juglone exhibited beneficial effects in MS-PAH rats in this short treatment period, prolonged use of juglone might be toxic [48]. In the current study, we provide proof of principle showing that Pin1 inhibition might be beneficial in experimental PAH. Arguably, selective inhibition of Pin1 with a more specific inhibitor, with a prolonged time period may even result in a stronger reversal of PAH. Although no side effects were observed in these preclinical models, future studies should test novel selective inhibitors of Pin1 in combination with other PAH drugs and also should aim to develop lung-specific delivery methods[49] to achieve efficient efficacy at low concentrations.

In summary, we provide evidence that Pin1 plays a role in inducing EC dysfunction and thereby promotes adverse vascular remodelling in PAH. Inhibition of Pin1 reduces proliferation, inflammation and TGF-β signalling, and augments BMP signalling. We conclude that inhibition of Pin1 displays beneficial effects in vitro and in vivo, and the development of a more selective Pin1 inhibitor might be beneficial (either alone or in combination with existing therapeutic approaches) for treating this deadly disease PAH.

## Supplementary Information

Below is the link to the electronic supplementary material.

Supplementary file1 (PDF 10389 kb)

Supplementary file2 (PDF 433 kb)

Supplementary file3 (PDF 120 kb)

Supplementary file4 (PDF 156 kb)

Supplementary file5 (DOCX 36 kb)

## Data Availability

The datasets generated during and/or analysed during the current study are available from the corresponding author on reasonable request. (data transparency).

## Code availability

(Software application or custom code).
