# Supplementary figures and images for: Inhibition of the prolyl isomerase Pin1 improves endothelial function and attenuates vascular remodelling in pulmonary hypertension by inhibiting TGF-β signalling
Source: Angiogenesis. 2021 Aug 11;25(1):99–112. doi: 10.1007/s10456-021-09812-7 (PMC8813847; doi:10.1007/s10456-021-09812-7)

Figure S1

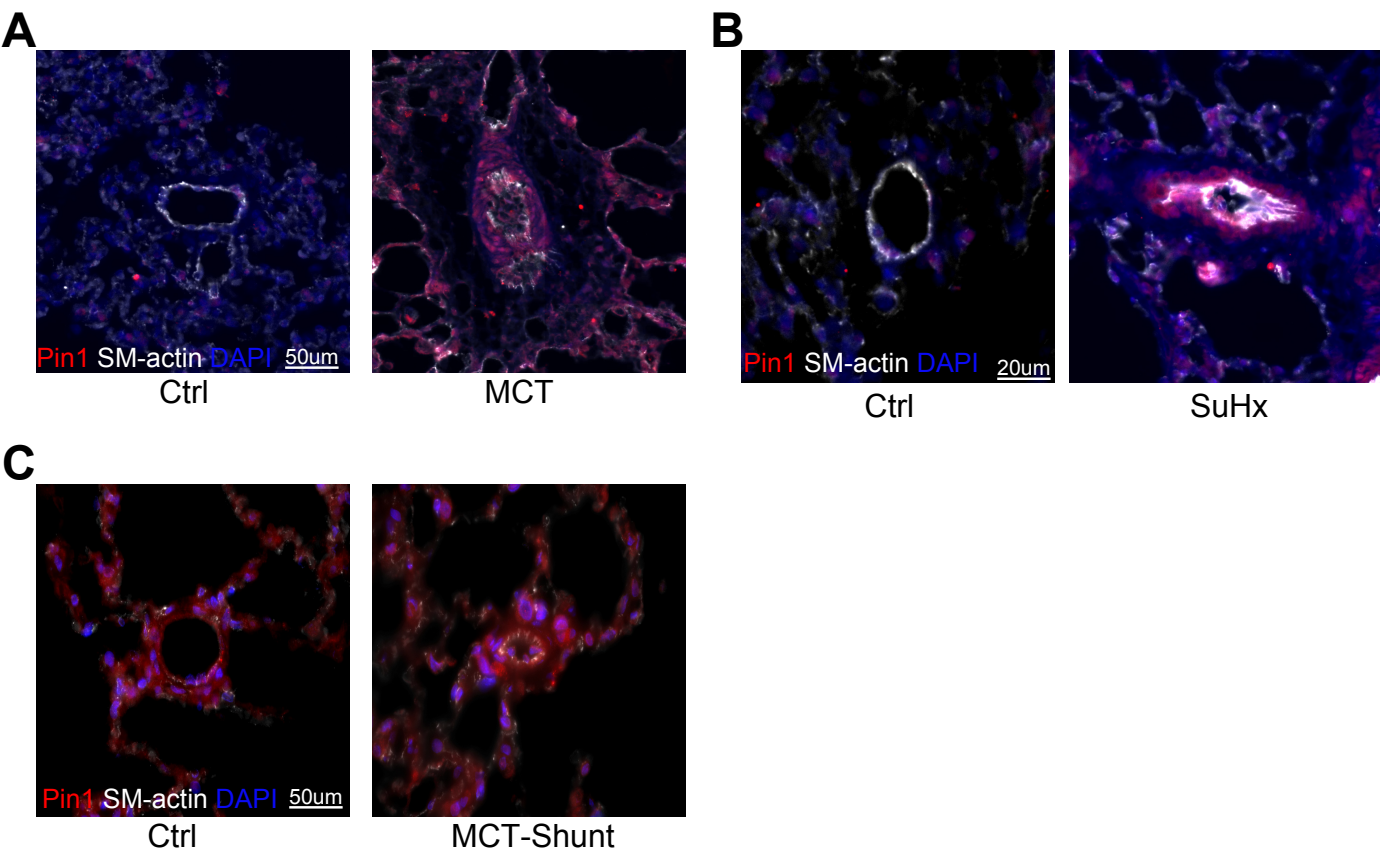

Supplement: Supplementary file 1 — Supplementary file1 (PDF 10389 kb) [file 10456_2021_9812_MOESM1_ESM.pdf]

Figure S2

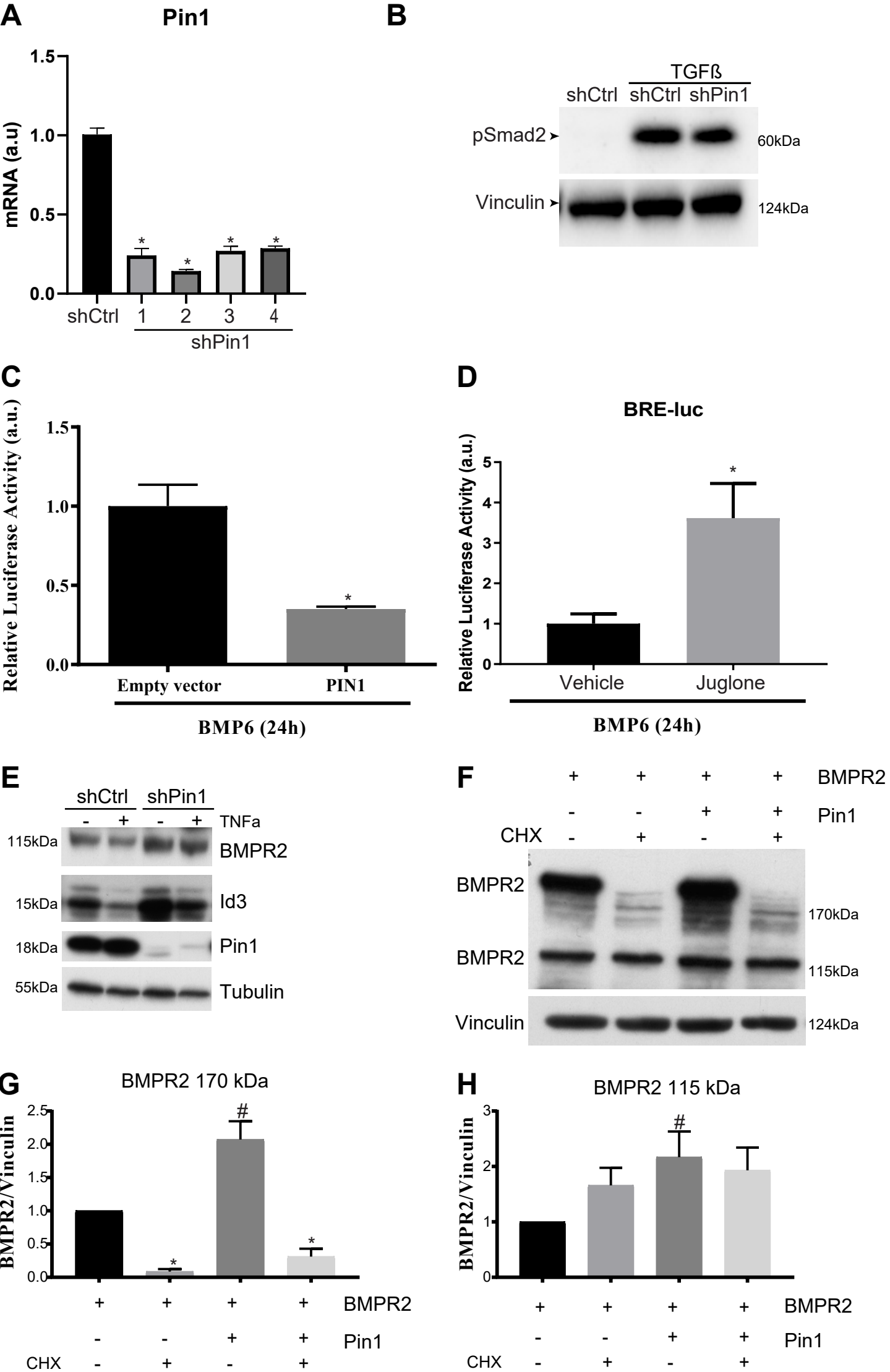

Supplement: Supplementary file 2 — Supplementary file2 (PDF 433 kb) [file 10456_2021_9812_MOESM2_ESM.pdf]

Figure S3

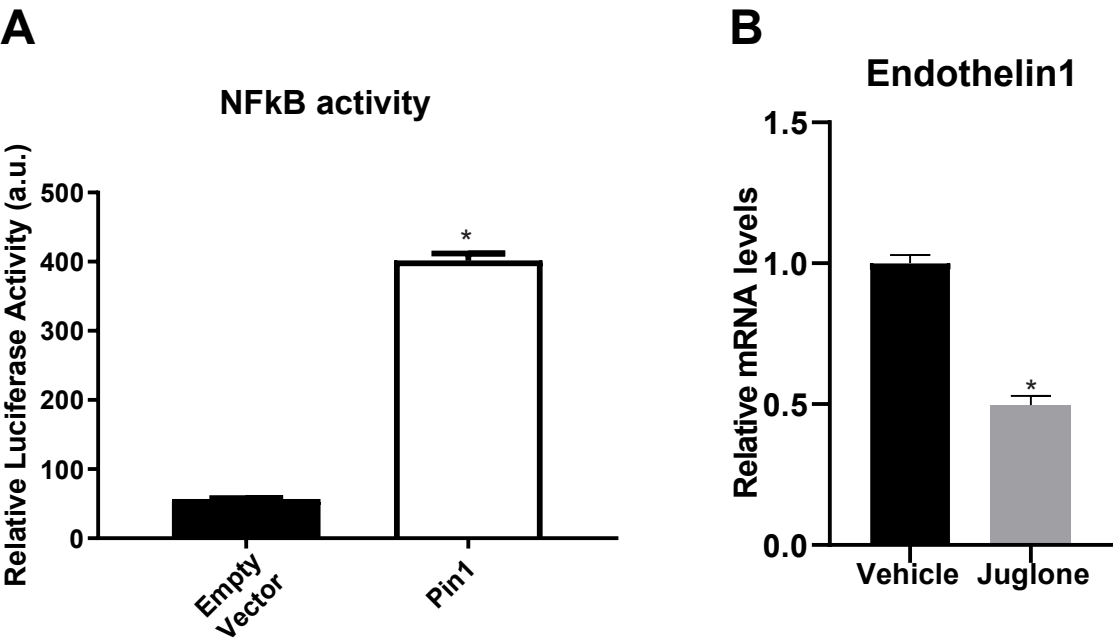

Supplement: Supplementary file 3 — Supplementary file3 (PDF 120 kb) [file 10456_2021_9812_MOESM3_ESM.pdf]

Figure S4

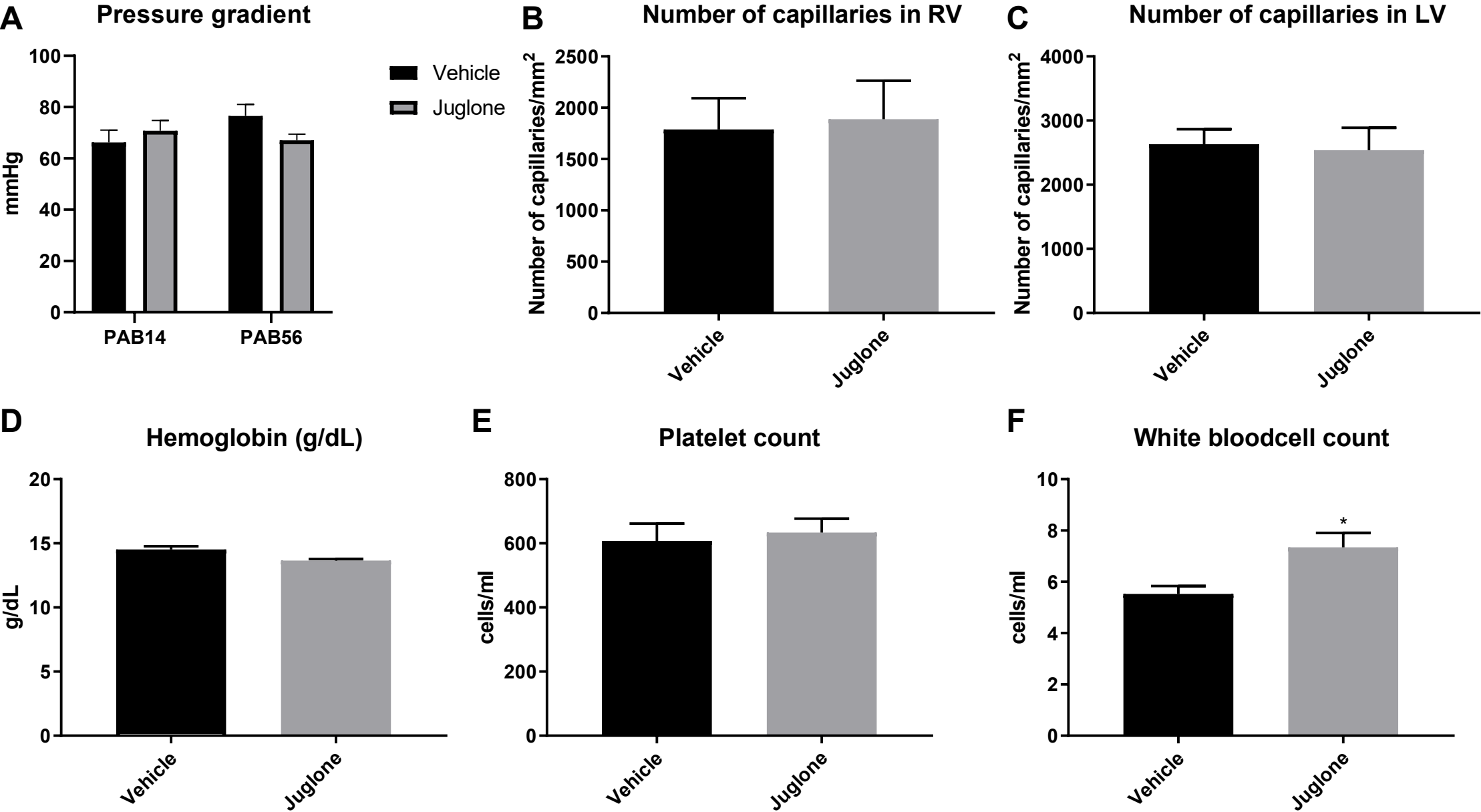

Supplement: Supplementary file 4 — Supplementary file4 (PDF 156 kb) [file 10456_2021_9812_MOESM4_ESM.pdf]
