# Supplementary material for: Inhibition of the prolyl isomerase Pin1 improves endothelial function and attenuates vascular remodelling in pulmonary hypertension by inhibiting TGF-β signalling
Source: Angiogenesis. 2021 Aug 11;25(1):99–112. doi: 10.1007/s10456-021-09812-7 (PMC8813847; doi:10.1007/s10456-021-09812-7)
Supplement: Supplementary file 5 — Supplementary file5 (DOCX 36 kb) [file 10456_2021_9812_MOESM5_ESM.docx]

**Inhibition of the prolyl isomerase Pin1 improves endothelial function and attenuates vascular remodelling in pulmonary hypertension by inhibiting TGF-β signalling**

Kondababu Kurakula^1^, Quint A.J. Hagdorn^2^, Diederik E. van der Feen^2^, Anton Vonk Noordegraaf^3^, Peter ten Dijke^4^, Rudolf A. de Boer^5^, Harm Jan Bogaard^3^, Marie-José Goumans^1*^, Rolf M.F. Berger^2*^

^1^Department of Cell and Chemical Biology, Leiden University Medical Center, Leiden, The Netherlands. ^2^Center for Congenital Heart Diseases, Department of Paediatric Cardiology, Beatrix Children’s Hospital, University Medical Center Groningen, University of Groningen, the Netherlands. ^3^Department of Pulmonary Medicine, Amsterdam Cardiovascular Sciences, Amsterdam UMC, Vrije Universiteit Amsterdam, Amsterdam, The Netherlands.^4^Department of Cell and Chemical Biology, Oncode institute, Leiden University Medical Center, Leiden, The Netherlands. ^5^Department of Cardiology, University Medical Center Groningen, University of Groningen, Groningen, The Netherlands.

*These two authors jointly supervised this work.

**Supplemental methods:**

**Cell culture and tissue sections**

Collection of lung specimens was approved by the local ethical committee and written informed consent from patients was obtained. Human pulmonary artery microvascular endothelial cells (MVECs) and smooth muscle cells (PASMCs) were isolated and cultured from idiopathic PAH patients and control lung explant tissue as previously described.[1] Cells between passage 5 and 8 were used for experiments. Lung tissue sections from untreated monocrotaline (MCT) and Sugen-hypoxia (SuHx) induced PH rat models were used, as described previously.[1, 2] HEK293T cells were cultured in Dulbecco's modified Eagle's medium (DMEM) with 20 mM glucose, supplemented with 10% serum and penicillin-streptomycin (Invitrogen).

**Quantitative Real Time-PCR (qRT-PCR)**

Total RNA from cultured cells was isolated using NucleoSpin RNA II isolation kit (Machery Nagel, Düren) according to the manufacturer’s instructions. Total RNA from rat lung and RV tissue was isolated using TRIPURE (Roche). All the cDNA was synthesized with the iScript cDNA kit (Bio-Rad) following the manufacturer's instructions. RT-qPCR was performed with SYBR Green Supermix (Bio-Rad) using specific primers on CFX Real-Time PCR detection System (Bio-Rad). Analysis of qPCR results was done using the delta delta Ct method. GAPDH was used as an internal control for the amount of cDNA present in each sample. Primers used for real-time PCR are detailed in supplementary table 1.

**Western blot**

Whole cell and lung lysates were prepared with NP-40 lysis buffer containing phosphatase and protease inhibitors (Sigma-Aldrich), and western blotting was performed as described.[1] Briefly, equal amount of protein was resolved by SDS-PAGE and transferred to polyvinylidene difluoride (PVDF) membranes (Millipore). Blots were blocked with 5% non-fat dry milk in PBS/TBS with 0.1% Tween 20 (PBST/TBST) for 1 hour at RT and incubated with primary antibodies overnight at 4°C. After washing with TBST buffer, membranes were incubated with appropriate horseradish peroxidase (HRP)-conjugated anti-mouse or anti-rabbit (GE Healthcare) secondary antibodies for 1 hour at RT in 5% non-fat milk. Antibodies were revealed using an ECL system (Fisher Scientific) and labeled proteins were detected with the imaging Chemidoc MP system (Bio-Rad). Protein expression was quantified using Image J software and normalized to GAPDH or Vinculin as previously described. The following antibodies directed against Pin1 (1:500; Proteintech, 10495-1-AP), pSmad2/3 (1:1000; pS2 antibody), pSmad1/5/8 (1:1000; pS1 antibody)[3], BMPR2 (1:1000; Thermo Scientific, MA5-15827), ID3 (1:200; Santa Cruz, sc-490), and Vinculin (1: 1000; H300; Santa Cruz) were used.

**Cell viability assays**

Cell viability was determined by 3-(4,5-dimethyl-2-thiazolyl)-2,5-diphenyl-2H-tetrazolium bromide (MTT) assays and were performed as described previously.[4] Briefly, cells were seeded in a 96-well plate at a density of 3×10^3^ cells/well in ECM medium (Sciencell, Catalog#1001) and incubated overnight. Cells were made synchronized by incubation in medium without FCS for 6 h and then incubated overnight with FCS (10% [vol/vol]) and vehicle or juglone (1uM) for 24 h. After the incubation time, 10μl of MTT reagent (5 mg/ml) was added to the cells for 3 h at 37°C. 100μl of isopropanol was added to each well and incubated for 15 min at RT. Colorimetric analysis was performed with a spectrophotometer. Each experiment (in quadruplicate) was repeated at least three times.

**Cell count analysis**

MVECs and PASMCs were pretreated with vehicle or juglone (1uM) for 48h and the number of cells was counted using a TC20™ Automated Cell Counter (Bio-Rad).

**Determination of PASMC growth in cultured medium of MVECs**

PAH MVECs were pretreated with vehicle or juglone (1uM) for 24 h. Conditioned medium from these cells was added to control PASMCs and incubated for 48 hours after which the number of cells were counted using TC20™ Automated Cell Counter (Bio-Rad). Of note, the half-life of juglone is 2 hours.

**Transient transfection and luciferase assays**

HEK293T cells were transiently transfected with the indicated luciferase reporter plasmids in the absence or presence of Pin1 using PEI transfection reagent (Polysciences), following the recommendations of the manufacturer. Primary MVECs were transfected using Lipofectamine 3000 (Thermo Scientific) as per the instructions of the manufacturer. Renilla plasmid was co-transfected as an internal control in both HEK293T cells and MVECs. 48 hours post-transfection, cells were harvested and lysed to measure the luciferase activity using the dual luciferase reporter assay system (Promega) by a Perkin Elmer luminometer Victor3 1420. An empty vector was used to equalize the total amount of plasmid DNA in each well when necessary and each experiment (in triplicate) was repeated at least three times.

**Plasmids and chemicals**

Pin1 over-expression plasmids, luciferase reporter constructs: BMP response element (BRE)-luc, TGF-β–responsive CAGA-luc, CyclinD1-luc, and NFκB-luc have been described before.[1, 5] TNFα (Peprotech; cat#300-01A) and TGFβ3 ( 8420-B3, R&D SYSTEMS and Andrew P. Hinck, University of Pittsburg, USA) were used. Lentiviral shRNA plasmids targeting Pin1 [shPIN1#1-5] were ordered from a shRNA library (shPin1-1: TRCN0000010577, shPin1-2: TRCN0000001036, shPin1-3: TRCN0000001033, shPin1-4: TRCN0000001034, shPin1-5: TRCN0000001035; Sigma-Aldrich, Cat No: SHCLND-NM_006221, Missouri, USA).

**Lenti-viral transduction**

Recombinant lentiviral particles of overexpression of Pin1 and shRNAs targeting Pin1 were produced, concentrated, and titrated as described previously.[1] Five different human shRNAs that target different regions in the Pin1 mRNA were tested and two shRNAs (TRCN0000010577, TRCN0000001036) were chosen for generation of lentiviruses based on the efficiency of the knockdown. Lentiviral transduction in cultured MVECs was performed as described previously.[1] Knockdown efficiency was determined by qRT-PCR and western blot.

**Juglone treatment in the MCT+Shunt rat PAH model**

All animal experiments were approved by the Dutch Central Ethical Committee for Animal Experiments and the Animal Care Committee of the University Medical Center Groningen and were carried out in compliance with guidelines issued by the Dutch government (permit numbers AVD105002015129 and AVD105002015134). All experiments were conducted according to published standards for preclinical and translational research in PAH.[6] The MCT+Shunt rat PAH model was used to study pulmonary vascular remodeling and was performed as described previously.[7] Briefly, 25 Wistar male rats (±200g; Charles River, Fr) were kept in a 12-hour light∶dark cycle and received standard rodent food and water ad lib. Flow-induced PAH was induced by a subcutaneous 60 mg/kg injection of monocrotaline (MCT, Sigma) at T0 days, followed at T7 by aorto-caval shunt surgery, which approximately doubles pulmonary blood flow. MCT+Shunt (MS) induces neomuscularization and medial hypertrophy from T7 to T14, neointimal lesions from T14 to T21 and RV failure from T21 to T28. Rats were randomly assigned to 3 groups: 1) MCT+Shunt sacrificed at T21 (MS21) as a baseline group; 2) treatment with vehicle (5% DMSO in drinking water) from T21, with sacrifice at T35 (MS35Veh); 3) treatment from T21 with 5mg/kg juglone in vehicle (5%DMSO in drinking water) with sacrifice at T35 (MS35Juglone). Echocardiography was also performed before the treatment(at day 21) started to determine baseline cardiac function. At the end of the study period, before sacrifice the animals, rats were anesthetized and subjected to hemodynamic evaluation by echocardiography and right heart catheterization. The lungs and heart were collected for further analysis. All measurements and analyses were done in a blinded manner.

**Juglone treatment in the PAB rat RV pressure load model**

In models of PAH, it is difficult to dissect whether effects of therapy on the RV result from a direct therapeutic effect on RV myocardium, or an indirect effect caused by modulated afterload. Therefore, to assess direct myocardial effects of juglone in this setting, isolated RV pressure load was created in 16 adult male Wistar rats (±200g, Charles River, Fr) by main pulmonary artery banding (PAB) surgery at T0, with a tight constriction at the size of an 18G needle. One rat died within the first hour after surgery. One rat was excluded due to a non-significant degree of pressure load, defined as a minimum systolic PAB gradient of 40mmHg on echocardiography at day 14. All rats showed to be subjected to effective RV pressure load (defined as a minimum systolic pressure difference of 40mmHg). PAB gradient was assessed by means of echocardiography at T14, where after rats were grouped into two groups with equal mean PAB gradient. Rats were randomly assigned to 1) treatment with vehicle (5% DMSO in drinking water) from T28 with sacrifice at T56 (PABveh56), or 2) treatment with 5mg/kg Juglone in vehicle (5% DMSO in drinking water) from T28 with sacrifice at T56. Before sacrifice, all rats underwent haemodynamic evaluation by echocardiography, after which lungs and hearts were collected for histopathologic evaluation. To assess the extent of RV hypertrophy, the RV free wall was separated from the left ventricle (LV) and ventricular septum. Wet weights of the RV, free LV and septum were determined separately, and the ratio of RV weight to LV plus interventricular septum weight (Fulton index: RV/[LV+IVS]) was calculated for RV hypertrophy. For further analysis of RV dimensions, 5-µm-thick paraffin sections of cardiac tissues were stained with haematoxylin and eosin, and a mean transversal cardiomyocyte cross-sectional area (CSA) was assessed.

**Quantitative pulmonary vascular morphometry**

5 µm paraffin-embedded lung sections were stained according to the Elastica-Van Gieson protocol and scanned on 40x magnification (Hamamatsu nanoviewer, Japan). All slides were masked, blinding the investigator for the experimental group. 40 vessels (diameter <50 µm) per lung were analyzed according to a standardized pulmonary vascular morphometry protocol, described in detail previously.[7, 8]

**Measurement of RV fibrosis**

For measurement of tissue fibrosis, 5 µm paraffin-embedded RV sections were stained according to the Masson protocol and scanned on 40x magnification (Hamamatsu nanoviewer, Japan). Then, % fibrosis was calculated using the positive pixel count algorithm (V9) on image scope V12.3.

**Immunofluorescence (IF) and immunohistochemistry (IHC)**

Paraffin-embedded lung or RV tissues were sectioned at 5µm, deparaffinized, rehydrated in graded washes of xylene to ethanol, and then subjected to heat-induced epitope retrieval using citrate buffer. Following antigen retrieval, the sections were blocked with 1% BSA in 0.1% Tween-PBS for 1h at room temperature. Then, sections were incubated with primary antibodies at 4°C overnight. Hematoxylin and eosin staining was performed for the morphometric assessment of pulmonary vessels. All sections were mounted with ProLong® Gold antifade reagent (Invitrogen) containing DAPI. Rabbit polyclonal anti-Ki67 (1:400, Millipore, ab9260), rabbit polyclonal anti-cleaved Caspase 3 (1:100, Cell Signaling, #9661), mouse monoclonal anti-alpha smooth muscle actin (αSMA, 1:200, Sigma, A2547), and rabbit polyclonal anti-αSMA (1:200, Abcam, ab5694) were used as primary antibodies. For IF, proteins were detected using appropriate fluorescent-dye conjugated secondary antibodies (Thermo Fisher Scientific). IF slides were analyzed under Zeiss AxioObserver Z1 inverted microscope. For IHC, species-specific immunoglobulin G coupled to peroxidase were used as secondary antibody and then stained by diaminobenzidine. All slides were masked and scanned on 40x magnification.

**Statistical analysis**

Statistical analyses were performed using the GraphPad Prism software for windows, version 7.0. The mean value (± SEM) was calculated for all samples, and significance was determined by either the unpaired t-test or analysis of variance (one- way ANOVA). Bonferroni multiple comparison test was applied to correct for multiple testing. A value of P < 0.05 was considered significant.

**Supplemental References**

1. Kurakula K, Sun X-Q, Happé C, et al (2019) Prevention of progression of pulmonary hypertension by the Nur77 agonist 6-mercaptopurine: role of BMP signalling Kondababu. Eur Respir J 1802400. https://doi.org/10.1183/13993003.02400-2018

2. da Silva Goncalves Bos D, Happe C, Schalij I, et al (2017) Renal Denervation Reduces Pulmonary Vascular Remodeling and Right Ventricular Diastolic Stiffness in Experimental Pulmonary Hypertension. JACC Basic to Transl Sci 2:22–35. https://doi.org/10.1016/j.jacbts.2016.09.007

3. Persson U, Izumi H, Souchelnytskyi S, et al (1998) The L45 loop in type I receptors for TGF-beta family members is a critical determinant in specifying Smad isoform activation. FEBS Lett 434:83–87. https://doi.org/10.1016/s0014-5793(98)00954-5

4. Kurakula K, Hamers AA, van Loenen P, de Vries CJM (2015) 6-Mercaptopurine reduces cytokine and Muc5ac expression involving inhibition of NFkappaB activation in airway epithelial cells. Respir Res 16:73. https://doi.org/10.1186/s12931-015-0236-0

5. Dennler S, Itoh S, Vivien D, et al (1998) Direct binding of Smad3 and Smad4 to critical TGF beta-inducible elements in the promoter of human plasminogen activator inhibitor-type 1 gene. EMBO J 17:3091–3100. https://doi.org/10.1093/emboj/17.11.3091

6. Provencher S, Archer SL, Ramirez FD, et al (2018) Standards and Methodological Rigor in Pulmonary Arterial Hypertension Preclinical and Translational Research. Circ Res 122:1021–1032. https://doi.org/10.1161/CIRCRESAHA.117.312579

7. van der Feen DE, Weij M, Smit-van Oosten A, et al (2017) Shunt Surgery, Right Heart Catheterization, and Vascular Morphometry in a Rat Model for Flow-induced Pulmonary Arterial Hypertension. J Vis Exp 1–11. https://doi.org/10.3791/55065

8. Van Der Feen DE, Kurakula K, Tremblay E, et al (2019) Multicenter preclinical validation of BET inhibition for the treatment of pulmonary arterial hypertension. Am J Respir Crit Care Med 200:910–920. https://doi.org/10.1164/rccm.201812-2275OC

**Supplementary Table 1: Primer sequences for qRT-PCR**

| Gene | Primers |
| --- | --- |
| Human Pin1 | Fw: CAGAGCGCGTCTAGCCAA  Rv: AGGAGCGTGGCCTAACTAGA |
| Human RANTES | Fw: CGCTGTCATCCTCATTGCTA  Rv: TGTACTCCCGAACCCATTTC |
| Human TNF-α | Fw: AGGACACCATGAGCACTGAAAG  Rv: AGGAGAGGCTGAGGAACAAG |
| Human PAI-1 | Fw: CACAAATCAGACGGCAGCACT  Rv: CATCGGGCGTGGTGAACTC |
| Human Id1 | Fw: CTGCTCTACGACATGAACGG  Rv: GAAGGTCCCTGATGTAGTCGAT |
| Human MCP-1 | Fw: CCTAGCTTTCCCCAGACACC  Rv: CCCAGGGGTAGAACTGTGG |
| Human GAPDH | Fw: AGCCACATCGCTCAGACAC  Rv: GCCCAATACGACCAAATCC |
| Rat VCAM-1 | Fw: TGTGGAAGTGTGCCCGAAAT  Rv: TGCCTTGCGGATGGTGTAC |
| Rat ICAM-1 | Fw: GGGCCCCCTACCTTAGGAA  Rv: GGGACAGTGTCCCAGCTTTC |
| Rat MCP-1 | Fw: ATGCAGTTAATGCCCCACTC  Rv: TTCCTTATTGGGGTCAGCAC |
| Rat GAPDH | Fw: GGTGGACCTCATGGCCTACA  Rv: TCTCTTGCTCTCAGTATCCTTGCT |

**Supplemental Figure Legends:**

**Figure S1: Pin1 expression is increased in the lungs of experimentally induced PAH rat models.** (A-C) Representative immunofluorescence photomicrographs of Pin1 (red) and α-smooth muscle actin (SM-actin, white) in the lungs from control and monocrotaline (MCT) (A), sugen hypoxia (SuHx) (B), and MCT-Shunt (C) induced PAH rat models. DAPI (blue).

**Figure S2: Pin1 modulates TGF-β/BMP signalling in vitro.** (A) Knock-down efficiency of Pin1 with lentiviruses encoding shRNA targeting Pin1 (shPin1#1-5) or a control shRNA (shCtrl) in pulmonary microvascular endothelial cells (MVECs) was determined by qRT-PCR. (B) Representative western blots showing pSmad2 in pulmonary artery smooth muscle cells (PASMCs) following knock-down of Pin1 and stimulation with TGFβ (1ng/ml) for 1 h. Vinculin served as a loading control. (C-D) BRE-luciferase activity in HEK293T cells was measured following over-expression of Pin1 (C) or treatment with Pin1 inhibitor, juglone (B) and stimulation with BMP6 (20ng/ml) for 24 h. (E) Representative western blots showing BMPR2, Id3 and Pin1 proteins relative to Tubulin as loading control following knock-down of Pin1 and stimulation with or without TNFα (50 ng/ml) for 6 h in MVECs. (F-H) HEK293T cells were transfected with expression plasmids coding for BMPR2 with or without Pin1. After 24h of transfection, the cells were treated with cycloheximide (CHX) to block the de novo protein synthesis for 16 h. BMPR2 expression was analysed by western blotting using anti-BMPR2 antibody (F) and quantified for BMPR2 170kDa (G) and BMPR2 115kDa protein. Vinculin was used as the loading control. *p<0.05 No CHX vs CHX treatment; #p<0.05 empty vector vs Pin1 overexpression.

**Figure S3: Pin1 modulates NFκB reporter activity and endothelin 1 expression.** (A) NFκB-luciferase activity in HEK293T cells was measured following over-expression of Pin1 and stimulation with TNFα (50 ng/ml) for 6 h. (B) qRT-PCR in MVECs was performed to assess mRNA expression of endothelin 1 following treatment with juglone for 6 h. *p<0.05. Student’s t-tests were used for comparisons between two groups. Error bars, mean ± s.e.m.

**Figure S4: Juglone effects on number of capillaries and blood cell composition in rats with RV pressure load induced by pulmonary artery banding (PAB) in vivo. (**A) Pressure gradient was measured in PAB rats at Day 14 and Day 56 following treatment with or without juglone. (B-C) The number of CD31 positive capillaries were counted in RV (B) and LV (C). The data is represented as number of capillaries/mm^2^. (D-F) Hemoglobin (D), platelets (E) and White-blood cells (F) were counted using coulter counter after terminating the rats. *p<0.05. Student’s t-tests were used for comparisons between two groups. Error b
